# A Psychometric Examination of the Abbreviated Version of the Parenting Styles Scale Using a Sample of Chilean Adolescents

**DOI:** 10.3390/children11060716

**Published:** 2024-06-12

**Authors:** José Luis Gálvez-Nieto, Karina Polanco-Levicán, Sonia Salvo-Garrido, María Pía Godoy-Bello

**Affiliations:** 1 Departamento de Trabajo Social, Universidad de La Frontera, Temuco 4780000, Chile; jose.galvez@ufrontera.cl; 2 Programa de Doctorado en Ciencias Sociales, Universidad de La Frontera, Temuco 4780000, Chile; 3 Departamento de Psicología, Universidad Católica de Temuco, Temuco 4780000, Chile; 4 Departamento de Matemática y Estadística, Universidad de La Frontera, Temuco 4780000, Chile; sonia.salvo@ufrontera.cl; 5 Departamento de Psicología, Universidad de La Frontera, Temuco 4780000, Chile; mariapia.godoy@ufrontera.cl

**Keywords:** parenting styles, parental socialization, adolescence

## Abstract

Parenting styles have been related to a series of variables that contribute positively to adulthood. The maternal and paternal parenting styles scale is a measure that presents a multidimensional structure of six correlated factors. However, the version available for Chile is extensive, with 82 items measuring this latent trait. Therefore, the aim of this study was to examine the psychometric properties of the reliability and validity of the abbreviated version of the maternal and paternal parenting styles scale using a sample of Chilean adolescents. The sample consisted of 2131 students of both the male and female sexes (51% males and 49% females) with a mean age of 15.85 years (SD = 1.37). The results of the exploratory and confirmatory factor analyses supported the six-factor correlated model, showing robust psychometric indices for both modelling approaches. In relation to the factorial invariance models, the results show factorial equivalence at the scalar invariance level for the variables of sex, age, academic achievement, and type of school. The scale showed adequate levels of reliability. This study concludes that the abbreviated version of the maternal and paternal parenting styles scale is a reliable and valid instrument for its application in Chilean adolescents.

## 1. Introduction

Parenting styles continue to be the focus of attention due to their cognitive, emotional, relational, and behavioural implications, in addition to their relevance throughout life, particularly in adolescence, considering that they can be protective factors or risk factors at this stage [1,2,3,4]. Parenting styles influence the socialization of children [5,6,7,8]. In adolescence, fathers and mothers continue to have fundamental roles in parenting; although these roles are transformed with respect to previous stages, children benefit from adults who are affectionate, communicative, and close [5,6,7,8]. Positive parenting styles, i.e., interactions that are beneficial for children, influence emotion regulation abilities [7] and life satisfaction in adolescents, decreasing, for example, externalizing behaviours [6], which are associated with the authoritative parenting style. Parenting styles of a negative nature are linked to difficulties in different areas of children’s lives, such as problematic internet use [9,10] and suicidal thoughts in adolescence [11,12], which are associated with a neglectful parenting style. Therefore, it is necessary to work with parents or adults who raise and educate adolescents [13], for which it is essential to have valid and reliable scales in the adolescent population in order to make evaluations for research and intervention.

Parental socialization allows children to become adults who can perform adequately in society based on parental role models in different experiences and situations. Thus, it is important that children acquire habits, skills, and values, among other factors, considering that the family is the main context of socialization [14,15]. Now, parents have different ways of interacting, that is, different ways of exercising control and showing affection [14,16]. Evaluating parenting styles allows us to comprehensibly organize how parents socialize their children by considering different parental practices [14,17,18]. Parenting styles are the attitudes manifested in a stable manner in the interactions between parents and their children that generate a particular emotional climate [19]. Baumrind [16] proposed three types of parenting: authoritarian, authoritative, and permissive. Later, Maccoby and Martin [20], based on the study by Baumrind [16], proposed four parenting styles that are differentiated according to the combination of affection and control, namely authoritative (high control/high affection), negligent (low control/low affection), permissive (low control/high affection), and authoritarian (high control and low affection), which allow us to develop a better understanding of the permissive style and differentiate it from relationships that have little affection and little control on the part of the parents.

In the same line, from the typological approach that combines control and affection, different investigations have been carried out, showing that the authoritarian style is associated with poor school performance, since the excess of rules and demands and the student’s lack of autonomy, affection, and parental support that is necessary to face difficulties hinders their achievements [21]. In addition, this style is associated with significant levels of aggression in the short and long term [3]. This can be a risk factor for Internet addiction and addiction to online gaming [1]. Along the same lines, the neglectful parenting style is related to the aggressor–victim role in bullying [22]. On the other hand, authoritative parenting is indirectly linked to self-motivation for physical activity through parental practices related to physical exercise [23]. Also, this parenting style is shown to be less related to children’s internalizing or externalizing problems [24]. Meanwhile, indulgent parenting is associated with lower drug use and better psychosocial adjustment scores [2], and results in adolescents who are more confident, trusting, and tolerant of their peers [25]. Indulgent and authoritative families have adolescents with higher self-efficacy perceptions [26]. In addition, they can be considered protective factors against Internet addiction and online gaming addiction [1]. When comparing parenting styles by sex, authors such as Capano et al. [27] identified that daughters perceived their fathers as less affectionate and rational compared to the perceptions held by sons; in contrast, no significant differences were reported in sons’ nor daughters’ perceptions of mothers.

On the other hand, a dimensional approach was proposed that not only considers affect and control as fundamental characteristics in parenting styles, but also integrates other important variables to be evaluated in the relationship between parents and children, allowing the possibilities of understanding this phenomenon to be broadened and making the analysis of parenting styles more complex. The above approach aims to promote a family environment conducive to the proper development of adolescents through positive parenting [28,29,30]. In this sense, communication, psychological and behavioural control, autonomy promotion, self-disclosure, and humour emerge as characteristics that occur in parental relationships that influence various areas [28,29,30]. Consequently, styles prioritizing affection, communication, and autonomy are related to positive parenting outcomes [6]. Furthermore, families that support autonomy promote engagement in learning in adolescents [31]. Warmth and support in parenting promote behavioural safety and psychological resilience in adolescents [32,33,34]. Also, self-disclosure in adolescence allows parents to learn about their children’s experiences, activities, and behaviours [35,36], favouring the prevention of risky behaviours [37]. In this sense, self-disclosure scarcely occurs in rejecting and indifferent families, and it has been observed that victims of bullying who present suicidal ideation do not inform their parents [38].

Regarding the dimensions related to control and its short- and long-term effects on adolescent development, it can be noted that behavioural control decreases internet addiction. In contrast, parental psychological control was a predictor of internet addiction in adolescents in a period of over three years [39]. In a study that spanned two years, parental psychological control was reported to be associated with peer victimization in adolescent students [5]. In another longitudinal study, it was observed that adolescents who report lower levels of parental psychological control also manifest a greater sense of autonomy during a two-year follow-up [40]. In cross-sectional studies, there is evidence that psychological control is associated with a frequency of panic symptoms among adolescents [41], with increased internalizing and externalizing problems [42], affecting parent–child closeness [32]. According to Gorostiaga et al. [43], parental warmth, behavioural control, and autonomy promotion are negatively related to internalizing symptoms in adolescents; in contrast, psychological control is linked to anxiety, depression, and suicidal tendencies in adolescents.

It is important to note that the parenting style may differ from the mother to the father, as it was observed that fathers may be perceived as more authoritarian compared to mothers, who are perceived as authoritative, and that both maternal and paternal authoritative parenting styles were positively associated with life satisfaction [44]. Also, a father’s rejecting behaviour is directly and significantly associated with the tendency to react angrily to various situations. At the same time, control on the part of the mother would influence symptoms of depression and anxiety [45]. Along the same line, a mother’s authoritarian style is associated with suicidal ideation, with this relationship being moderated by the mother’s assertiveness [46], while a father’s authoritarian style is related to mental health problems in their children [47]. However, if the mother’s and father’s behaviours coincide in demonstrating greater warmth and lower demands, their children show fewer mental health problems [48].

Considering the differences between the parenting style and the dimensional approach, Oliva et al. [28] proposed three parenting styles called democratic, strict, and indifferent based on the combination of different dimensions present in parenting, such as humour, self-disclosure, behavioural and psychological control, and affection. Consequently, he proposed an instrument with six dimensions (affection and communication, behavioural control, psychological control, autonomy promotion, self-disclosure, and humour). Specifically, the democratic style involves the presence of affection in interactions in addition to parents promoting autonomy, manifesting good humour, and little psychological control and propitiating adequate disclosure. The strict style shows a high level of psychological and behavioural control and less affection than the democratic style, but self-disclosure, humour, and the promotion of autonomy are present. Finally, the indifferent style is characterized by fathers and mothers whose relationship with their children shows low levels in the indicated dimensions; however, higher scores predominate in psychological control [49]. This scale was developed and applied to a sample of adolescents in Spain [28]; it has 82 items (41 items addressed to the mother and 41 items to the father). Subsequently, the abbreviated scale was presented, and the research was carried out in a Spanish sample whose results show six dimensions. However, the number of items was reduced to 24 [50], demonstrating adequate psychometric properties.

This instrument has been used by several researchers in both its long and short formats, showing its adequate psychometric properties [50,51,52]. According to Gómez-Ortiz et al. [49], the parental categories found were mostly located in the democratic style, i.e., they consider affection and communication in parenting, behavioural control, humour, as well as autonomy promotion, with the affection/communication variable being the most relevant for adolescent adjustment [28]. Positive parenting styles are associated with participation in extracurricular activities [53] and with life satisfaction [54,55], specifically affect and communication, autonomy promotion, self-disclosure, and humour [30]. In addition, characteristics that promote autonomy, affection and communication, self-disclosure, and humour positively influence adolescents’ sports motivation [56]. It was added that adequate behavioural control and disclosure favour time devoted to study and academic performance [57]. Meanwhile, psychological control is associated with externalizing symptomatology [58]. It is considered a risk factor for internalizing problems [59], while greater affection, behavioural control, disclosure, and mood are linked to lower psychopathological symptoms [60]. Álvarez-García et al. [50] pointed out that the greater the affection, communication, autonomy, behavioural control, humour and disclosure, the lower the offline school aggression and antisocial behaviour. However, there are differences between maternal and paternal styles in relation to physical and verbal aggression, as they are influenced by the psychological control and autonomy promotion shown by parents [61].

Given the above, it is important to point out the relevance of addressing parenting styles considering the influence they have on different aspects of adolescents’ lives at the social, emotional, and cognitive levels, which transcend the different stages of a person’s life [30,56]. Therefore, assessing parenting styles is fundamental to supporting fathers and mothers in favouring adolescents’ adaptation as it could decrease emotional and behavioural problems [62]. On the other hand, it is important to mention that parenting styles and their results in different settings may vary depending on the culture [63]. Thus, in some cultures, better results of the permissive style are appreciated [26]. Therefore, validating this scale in its abbreviated version in Chilean adolescents is a contribution to the existing literature.

Given parenting styles’ theoretical and empirical relevance, the following hypotheses are proposed: First, the scores obtained by the parenting styles scale are expected to confirm a factorial structure of six correlated factors with adequate levels of reliability. Second, the parenting styles scale scores will present levels of scalar invariance according to the variables of sex, age, and academic performance. Consequently, this research examines the psychometric properties of the reliability and validity of the abbreviated version of the maternal and paternal parenting styles scale in a sample of Chilean adolescents.

## 2. Materials and Methods

### 2.1. Participants

A population of 486,427 adolescent students from public, charter, and private high schools in Chile (N) was investigated. A stratified multistage probability sample was chosen with a reliability of 99.7%, a margin of error of 3%, and a variance of p = q = 0.5 [64]. The sample consisted of 2131 students from 32 educational institutions, with both the male and female sexes (49% female), with an average age of 15.85 (SD = 1.37).

### 2.2. Instruments

A questionnaire was created to capture the demographic data of the sample. A set of closed questions was administered, e.g., gender, age, academic performance, and type of school.

In addition, the abbreviated parenting styles scale [50] was applied. This instrument was adapted from Oliva et al.’s [28] parenting styles scale. The abbreviated parenting style scale has 24 items divided into six factors: affection and communication (e.g., When I speak with my parents, they show interest and pay attention), the promotion of autonomy (e.g., My parents encourage me to think independently), behavioural control (e.g., My parents set a curfew for me), psychological control (e.g., My parents continuously try to monitor the way I am and think), self-disclosure (e.g., I tell my parents what I do in my free time), and humour (e.g, My parents are almost always cheerful and optimistic people). This instrument presents adequate psychometric properties in the Spanish population [50].

### 2.3. Procedure

School principals were contacted, and permission to administer the questionnaires was requested. The ethical principles of the participants were safeguarded, and informed consent was requested from mothers, fathers, or guardians and students. The study was approved by the Ethics Committee of the Universidad de La Frontera (ethics protocol number 034-19). Questionnaires were answered anonymously during the first period.

Regarding the scale adaptation process, it is relevant to mention that this instrument was originally published in Spanish, as well as the abbreviated scale [50] applied in this research. Therefore, it was not necessary to translate the scale. Subsequently, the instrument was reviewed by experts in different areas (methodologists, theoreticians, university professors, and students), concluding that no changes were required since the scale items should be adequately understood by the adolescents.

### 2.4. Data Analysis

Descriptive statistics were analysed for each item. Univariate and multivariate normality tests were evaluated to select the appropriate analysis approach. Subsequently, the sample was randomly divided into two equivalent halves. With the first sample, an exploratory factor analysis (EFA) was performed using the FACTOR software version 9.2 [65], using the Unweighted Least Squares Mean and Variance adjusted (ULSMV) estimation method, the polychoric correlation matrix, the Minimum Rank Factor Analysis extraction method [66], and an oblimin rotation. Subsequently, with the second half of the sample, using the MPLUS v.8.1 software [67], a confirmatory factor analysis (CFA) was applied to evaluate the scale structure using the polychoric correlations matrix and the ULSMV estimation method. The following goodness-of-fit indices were used to evaluate the CFA models: ULSMV-χ^2^, comparative fit index (CFI), Tucker–Lewis index (TLI), and root mean square error of approximation (RMSEA).

For CFI and TLI, values equal to or greater than 0.90 were considered reasonable [68]. For RMSEA, values less than or equal to 0.080 were considered a reasonable fit [69]. In addition, a factorial invariance analysis was conducted, including the following models [70]: M0 configural (equal number of factors), M1 metric invariance (equal factor loadings), and M2 scalar invariance (equal thresholds). The assessment of invariance was performed based on the following criteria [71,72,73]: ΔTLI, 0 = perfect and ≤ 0.01 = acceptable, and ΔRMSEA ≤ 0.015, as evidence of measurement invariance. For reliability estimation, using JASP v.012.2 software, the following coefficients were estimated: McDonald’s ω and Cronbach’s α [74,75].

## 3. Results

### 3.1. Descriptive Analysis

As shown in Table 1, the descriptive statistics of the 24 items show that item 9, “My parents try to know where I go when I go out”, presented the highest mean (M = 35.65; SD = 0.850). On the other hand, item 15, “My parents continuously try to control my way of being and thinking”, presented the lowest mean (mean = 2.72; standard deviation = 1.850). In addition, an assessment of univariate normality was conducted, where the results obtained through the Kolmogorov–Smirnov test indicated a rejection of the null hypothesis of normality (*p* < 0.001). In addition, an estimation of the multivariate kurtosis test was carried out, which agreed with the univariate tests in rejecting the hypothesis of multivariate normality (multivariate kurtosis coefficient = 62.183; *p* < 0.001).

### 3.2. Factor Structure

Once the data matrix was divided into two equivalent halves, we evaluated the relevance of performing an exploratory factor analysis. The results of the Kaiser–Meyer–Olkin index (KMO = 0.922) and Bartlett’s statistic (χ^2^ = 12159.6; *p* < 0.001) establish that the data matrix is appropriate for performing an EFA. A parallel analysis [76] suggested six factors that explain more variance than expected in random matrices. Together, these six factors explain 70.83% of the estimated variance. Table 2 shows the distribution of factor loadings for each of the six factors identified; these factors coincide with the original theoretical proposal.

**Figure 1:**
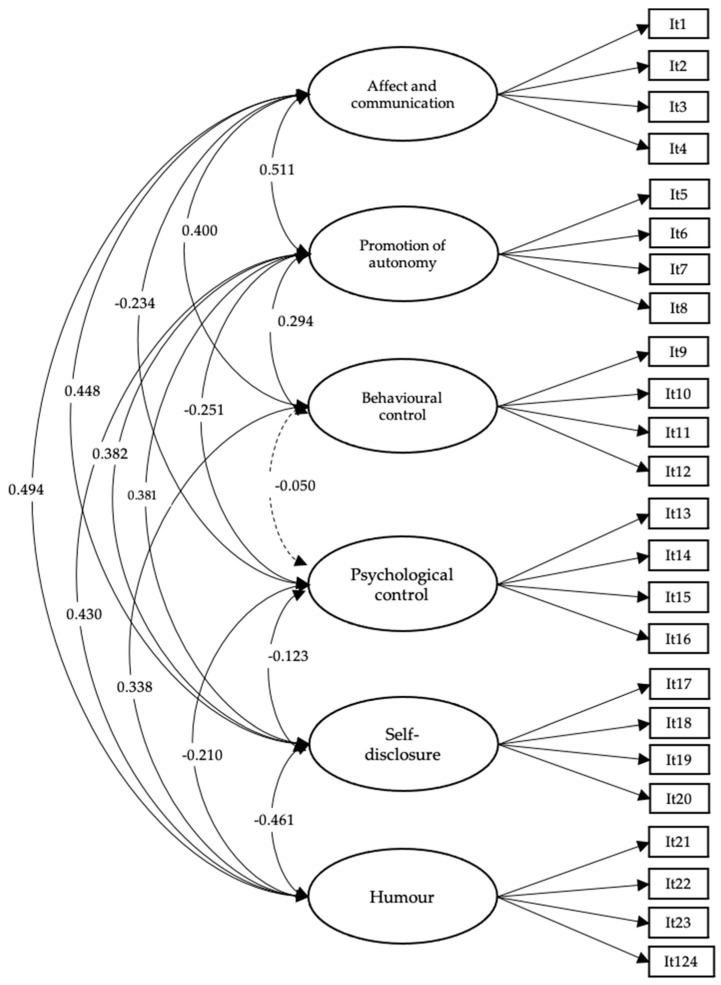
The factorial structure of the abbreviated parenting styles scale. All estimated parameters were statistically significant (*p* < 0.001).

Once the scale’s factor structure was identified, we proceeded to perform a CFA using the 24 items of the scale with the second half of the sample (Figure 1). As announced by the EFA, the six-factor correlated model showed satisfactory goodness-of-fit indices (ULSMV-χ^2^ (df = 237) = 829.031; CFI = 0.978; TLI = 0.974; RMSEA = 0.047 (CI = 0.043–0.050)). The results confirm that the original theoretical model of six correlated factors best fits the data.

In addition to the evidence of construct validity, the average variance extracted (AVE) was evaluated, and favourable results were obtained for all factors, namely humour (AVE = 0.75), behavioural control (AVE = 0.56), affection and communication (AVE = 0.72), promotion of autonomy (AVE = 0.67), self-disclosure (AVE = 0.63), and psychological control (AVE = 0.52), and an adequate value was obtained for the maximum shared variance discriminant validity (MSV = 0.62).

### 3.3. Factor Invariance

Once the factor structure of the scale was confirmed, measurement invariance analyses were conducted for gender (0 = male; 1 = female), academic achievement (0 = 1.0 to 4.0; 1 = 4.1 to 7.0), age (0 = 12 to 15 years; 1 = 16 to 20 years), and type of school (1 = public, 2 = charter, and 3 = private). Table 3 shows that the parenting styles scale reached a level of scalar invariance for all of the variables evaluated, suggesting equal thresholds.

### 3.4. Reliability Analysis

Table 4 illustrates the results of the reliability analysis. In general, all factors showed high reliability. The mood factor stands out with the highest level of reliability (ω = 0.886); in contrast, the behavioural control factor showed a slightly lower level (ω = 0.733).

## 4. Discussion

The general objective of this research was to examine the psychometric properties of the reliability and validity of the abbreviated version of the maternal and paternal parenting styles scale in a sample of Chilean adolescents. The results of this study allow us to support the complete fulfilment of this objective.

In relation to the evidence of validity, the results of this sample support that the factorial structure of the scale would present six correlated factors, which are consistent with the original proposal and have been named as follows: affect and communication, promotion of autonomy, behavioural control, psychological control, self-disclosure, and humour. These findings coincide with the first study that proposed this scale [28], which was applied to adolescents [50]. Furthermore, these results are in line with those reported by Álvarez-García et al. [50], who created the abbreviated version of the parenting styles scale. With respect to the reliability indices, the results, in general, show satisfactory values for each factor; these results are in line with previous studies [28,50,52].

Regarding the results of the measurement invariance models, the results show interesting findings. The measurement invariance was analysed for the variables of sex, academic achievement, age, and type of school. The results show that the scale is equivalent up to the scalar invariance level and reflect that the instrument measures without bias in all of the variables examined. This methodological contribution expands the results of previous research, which did not evaluate the equivalence of measurement according to individual and educational variables [28,50,52].

It is worth noting that the relevance of this research is related, firstly, to the fact that this construct (parenting styles) has captured the interest of researchers and professionals over time, as it has been linked to the proper development of adolescents. This research is relevant for families and society [1,3,4]. Secondly, the theoretical proposal behind the scale used in this research provides a more complex understanding and evaluation of parenting styles [28,29]. Thirdly, this instrument constitutes a contribution to studies in this area due to its ease of administration, encompassing various dimensions that refer to different characteristics of the interaction between parents and their adolescent children in a single instrument, which is psychometrically robust [28,29,50,52]. Therefore, this scale can be useful in intervention processes, allowing for comparisons to be made between the initial situation and possible changes at the end. It can be utilized by institutions and their professional teams working with families in the fields of health and education, among others. The above would allow for the development of adolescents to be promoted, bearing in mind that negative parenting styles contribute to various mental health difficulties, specifically internalizing and externalizing problems [5,42,46,58].

Despite the importance of this study, its results have certain limitations. The first is that the data come from a cross-sectional design and do not consider the influence of time on the measurement. In addition, the parenting styles scale is a self-report instrument, and this type of instrument presents problems due to the bias of self-assessment accuracy. It is important to note that convergent validity could not be estimated in this study through a scale that assesses the same construct or a theoretically related construct. Finally, the abbreviated scale presented does not consider the distinction between maternal and paternal parenting styles; however, the extended version for Chilean adolescents can be used [52].

Future research is expected to explore the validity and reliability of this scale in other cultures and different socio-demographic contexts, which will allow for a broader understanding of its psychometric characteristics across different population groups. Also, it would be relevant that this abbreviated version of the scale includes an adaptation for both fathers and mothers, considering that both may be perceived by their adolescent children with a different style. On the other hand, further research may explore the role of other variables that may mediate or moderate parenting styles and adolescent development. In this sense, considering the frequent use of the Internet and social networks, parenting styles could be related to Social Media Literacy (SML) by observing the association between the different dimensions of both constructs and evaluating their influence on the aggressions frequently occurring on the Internet.

## 5. Conclusions

This study demonstrates the validity and reliability of the abbreviated version of the maternal and paternal parenting styles scale in a sample of Chilean adolescents. The results support the factorial structure of six correlated factors, and they are consistent with the original proposal and with previous research. In relation to the contributions to knowledge, this research provides an instrument that allows for the assessment of the parenting styles construct with a shorter version than the original version of 82 items (41 for each parent), maintaining the six dimensions of the scale [50].

## Figures and Tables

**Table 1:** Descriptive statistics.

Items	M	SD	g1	g2	KS-Test
1.—Cuando hablo con mis padres. muestran interés y atención/When I talk to my parents, they show interest and attention.	5.20	1.09	−1.65	2.79	0.30 *
2.—Mis padres me animan a que les cuente mis problemas y preocupaciones/My parents encourage me to tell them about my problems and worries.	4.91	1.38	−1.34	1.09	0.26 *
3.—Si tengo algún problema puedo contar con su ayuda/If I have any problems, I can count on their help.	5.42	1.06	−2.25	5.13	0.39 *
4.—Mis padres muestran interés por mí cuando estoy triste y enfadado/a/My parents show interest in me when I am sad and angry.	5.14	1.24	−1.71	2.47	0.30 *
5.—Mis padres piensan que aunque aún no sea una persona adulta puedo tener ideas acertadas/My parents think I can have good ideas, although I am not yet an adult.	5.03	1.30	−1.54	1.86	0.28 *
6.—Mis padres me animan a que tome mis propias decisiones/My parents encourage me to make my own decisions.	5.02	1.30	−1.52	1.80	0.27 *
7.—Mis padres me animan a que piense de forma independiente/My parents encourage me to think independently.	5.13	1.21	−1.63	2.37	0.29 *
8.—Mis padres me permiten opinar cuando hay que tomar una decisión familiar/My parents allow me to have a say when a family decision must be made.	4.87	1.38	−1.28	0.90	0.24 *
9.—Mis padres intentan saber a dónde voy cuando salgo/My parents try to know where I go when I go out.	5.65	0.85	−3.26	12.10	0.45 *
10.—Si vuelvo tarde a casa, mis padres me preguntan por qué y con quién estuve/If I come home late, my parents ask me why and who I was with.	5.58	0.95	−2.96	9.48	0.43 *
11.—Mis padres ponen límites a la hora a la que debo volver a casa/My parents set limits on the time I have to go back home.	5.10	1.38	−1.64	1.83	0.33 *
12.—Mis padres me preguntan en qué gasto el dinero/My parents ask me what I spend my money on.	4.41	1.68	−0.86	−0.50	0.22 *
13.—Mis padres me hacen sentir culpable cuando no hago lo que quieren/My parents make me feel guilty when I do not do what they want.	3.27	1.89	0.131	−1.46	0.18 *
14.—Mis padres me dicen que ellos tienen razón y no debo llevarles la contraria/My parents tell me they are right and I should not contradict them.	4.02	1.76	−0.42	−1.16	0.18 *
15.—Mis padres intentan controlar continuamente mi forma de ser y de pensar/My parents constantly try to control the way I am and the way I think.	2.72	1.85	0.58	−1.17	0.25 *
16.—Mis padres dejan de hablarme cuando se enfadan conmigo/My parents stop talking to me when they get angry with me.	3.34	1.85	0.08	−1.42	0.16 *
17.—Les cuento a mis padres lo que hago en mi tiempo libre/I tell my parents what I do in my free time.	4.12	1.75	−0.57	−0.97	0.19 *
18.—Les hablo a mis padres sobre los problemas que tengo con mis amigos/as/I tell my parents about my problems with my friends.	3.90	1.88	−0.35	−1.35	0.18 *
19.—Cuando llego de la escuela, le cuento a mis padres cómo me ha ido el día/I tell my parents how my day went when I come home from school.	4.40	1.66	−0.80	−0.58	0.21 *
20.—Aunque no me pregunten, les cuento a mis padres cómo me va en las diferentes asignaturas/Even if they do not ask me, I tell my parents how I am doing in different subjects.	4.04	1.82	−0.50	−1.14	0.19 *
21.—Mis padres casi siempre son personas alegres y optimistas/My parents are almost always cheerful and optimistic people.	4.90	1.25	−1.25	1.18	0.23 *
22.—Mis padres suelen bromear conmigo/My parents often joke with me.	4.91	1.37	−1.31	1.01	0.26 *
23.—Es divertido hacer cosas con mis padres/It is fun to do things with my parents.	5.03	1.29	−1.48	1.73	0.28 *
24.—Mis padres se ríen mucho conmigo, My parents laugh a lot with me.	5.01	1.29	−1.41	1.40	0.28 *

M, Mean; SD, Standard Deviation; g1, Skewness; g2, Kurtosis; * *p* < 0.001.

**Table 2:** Factor loading matrix; exploratory factor analysis.

Item	F1	F2	F3	F4	F5	F6
It1	0.011	0.006	**0.740**	−0.002	0.021	−0.051
It2	−0.064	−0.022	**0.921**	−0.075	0.033	0.042
It3	−0.030	0.079	**0.692**	0.165	−0.019	−0.021
It4	0.054	−0.041	**0.833**	−0.022	0.022	0.013
It5	0.005	0.001	0.094	**0.624**	0.093	−0.056
It6	−0.012	0.001	0.061	**0.824**	−0.069	−0.038
It7	−0.023	0.019	−0.093	**0.936**	−0.033	0.039
It8	0.060	−0.049	0.109	**0.582**	0.045	0.070
It9	0.099	**0.801**	0.098	0.002	−0.155	−0.068
It10	−0.018	**0.897**	−0.009	0.034	−0.083	−0.033
It11	0.007	**0.726**	−0.056	−0.091	0.096	0.087
It12	−0.142	**0.587**	−0.078	0.064	0.159	0.081
It13	−0.025	0.047	−0.014	0.048	0.014	**0.693**
It14	0.118	0.045	0.065	−0.022	−0.120	**0.766**
It15	−0.098	−0.023	0.015	−0.077	0.082	**0.727**
It16	0.013	−0.061	−0.033	0.076	−0.027	**0.664**
It17	0.071	−0.006	−0.030	0.035	**0.735**	0.022
It18	−0.073	0.112	0.047	−0.017	**0.713**	−0.055
It19	0.070	0.003	0.135	−0.066	**0.683**	−0.014
It20	0.044	−0.086	−0.076	0.035	**0.777**	−0.010
It21	**0.551**	−0.036	0.110	0.039	0.092	−0.074
It22	**0.917**	0.004	−0.086	0.048	−0.043	0.059
It23	**0.853**	0.047	−0.091	0.024	0.091	−0.027
It24	**0.943**	−0.013	0.064	−0.083	−0.007	0.021

Note: F1: humour; F2: behavioural control; F3: affection and communication; F4: promotion of autonomy; F5: self-disclosure; F6: psychological control. Values in bold: factor loadings greater than 0.4.

**Table 3:** Measurement invariance.

Variable	Model	ULSMV −χ^2^ (df)	TLI	RMSEA	SRMR	ΔTLI	ΔRMSEA	ΔSRMR	Decision
Sex	Configural invariance	1431.050 (474)	0.961	0.044	0.035				Accepted
	Metric invariance	1303.808 (492)	0.968	0.039	0.036	0.007	0.005	0.001	Accepted
	Scalar invariance	1507.392 (582)	0.970	0.039	0.037	0.002	0	0.001	Accepted
Academic performance	Configural invariance	1334.245 (474)	0.962	0.043	0.035				Accepted
	Metric invariance	1232.863 (492)	0.969	0.039	0.035	0.007	−0.004	<0.001	Accepted
	Scalar invariance	1269.777 (582)	0.976	0.034	0.036	0.007	−0.005	0.001	Accepted
Age	Configural invariance	1302.410 (474)	0.963	0.041	0.034				Accepted
	Metric invariance	1160.618 (492)	0.971	0.036	0.036	0.008	−0.005	0.002	Accepted
	Scalar invariance	1178.703 (582)	0.978	0.031	0.035	0.007	−0.005	−0.001	Accepted
Type of school	Configural invariance	1498.180 (711)	0.980	0.039	0.039				Accepted
	Metric invariance	1501.509 (747)	0.982	0.038	0.040	0.002	0.001	0.001	Accepted
	Scalar invariance	1633.487 (879)	0.985	0.035	0.040	0.003	−0.003	0	Accepted

Note: ULSMV-χ^2^, unweighted least squares mean and variance-adjusted Chi-squared test; TLI, Tucker–Lewis index; RMSEA, root mean square error of approximation; SRMR, standardized root mean square residual.

**Table 4:** Reliability analysis.

Factors	McDonald’s ω	Cronbach’s α
Affection and communication	0.858	0.857
Promotion of autonomy	0.836	0.834
Behavioural control	0.733	0.724
Psychological control	0.757	0.756
Self-disclosure	0.819	0.819
Humour	0.886	0.884

## Data Availability

The dataset for the study is available from the corresponding author upon reasonable request due to ethical restrictions.
